# Cryopreserved human aortic root allografts arterial wall: Structural changes occurring during thawing

**DOI:** 10.1371/journal.pone.0175007

**Published:** 2017-04-17

**Authors:** Robert Novotny, Dasa Slizova, Jaroslav Hlubocky, Otakar Krs, Jaroslav Spatenka, Jan Burkert, Radovan Fiala, Petr Mitas, Pavel Mericka, Miroslav Spacek, Zuzana Hlubocka, Jaroslav Lindner

**Affiliations:** 1 2^nd^ Department of Cardiovascular Surgery, General University Hospital & 1^st^ Faculty of Medicine, Charles University, Prague, Czech Republic; 2 Department of Anatomy, Charles University- Faculty of Medicine in Hradec Kralove, Hradec Kralove, Czech Republic; 3 Transplant Center & Department of Cardiac Surgery, University Hospital Motol, Prague, Czech Republic; 4 Department of Cardiovascular Surgery, Faculty Hospital Motol & 2^nd^ Faculty of Medicine, Charles University, Prague, Czech Republic; 5 Tissue Bank, Faculty Hospital Hradec Kralove, Charles University- Faculty of Medicine in Hradec Kralove, Hradec Kralove, Czech Republic; 6 2^nd^ Internal Department of Cardiology and Angiology, General University Hospital & 1^st^ Faculty of Medicine, Charles University, Prague, Czech Republic; University of Milano, ITALY

## Abstract

**Background:**

The aim of our experimental work was to assess morphological changes of arterial wall that arise during different thawing protocols of a cryopreserved human aortic root allograft (CHARA) arterial wall.

**Methods:**

The experiment was performed on CHARAs. Two thawing protocols were tested: 1, CHARAs were thawed at a room temperature at +23°C; 2, CHARAs were placed directly into a water bath at +37°C.

**Microscopic samples preparation:**

After fixation, all samples were washed in distilled water for 5 min, and dehydrated in a graded ethanol series (70, 85, 95, and 100%) for 5 min at each level. The tissue samples were then immersed in 100% hexamethyldisilazane for 10 minutes and air dried in an exhaust hood at room temperature. Processed samples were mounted on stainless steel stubs, coated with gold.

**Results:**

Thawing protocol 1: All 6 (100%) samples showed loss of the endothelium and damage to the subendothelial layers with randomly dispersed circular defects and micro-fractures without smooth muscle cells contractions in the tunica media.

Thawing protocol 2: All 6 (100%) samples showed loss of endothelium from the luminal surface, longitudinal corrugations in the direction of blood flow caused by smooth muscle cells contractions in the tunica media with frequent fractures in the subendothelial layer

**Conclusion:**

All the samples thawed at the room temperature showed smaller structural damage to the CHARA arterial wall with no smooth muscle cell contraction in tunica media when compared to the samples thawed in a water bath.

## Introduction

Cryopreserved aortic root allografts (CHARA) have been used extensively in cardiac surgery for their advantages over bioprosthetic and mechanical valves, such as excellent hemodynamic function, very low thrombotic event rates, and mainly their resistance toward infections [1,2,3].

The era of allograft transplantation in cardiac surgery began after the first successful aortic valve transplantation performed by Ross in early 1962 based on Brewin experimental work [4,5]. The first allograft transplants in cardiac surgery were freshly harvested aortic valves that underwent minimal treatment with no ABO Blood group matching. Remarkably, these allograft transplants showed outstanding durability and performance, giving the basic foundation for this new type of surgical procedures. Due to do the lack of donors, cardiac centers started to treat allografts with antibiotics in order to prevent disease transmission, and cryopreserve them in order to prolong their life span. These techniques of allograft processing and cryopreservation led to significant decrease of allografts durability and their clinical performance between 1960s and early 1970s leading almost to the abandonment of this type of procedures [6].

The progress in allograft processing including cryopreservation had allowed the reintroduction of allograft transplants back into the cardiac surgery [2]. The major controversies reside in the issues of allografts durability and viability, which are strongly associated with allograft cryopreservation and thawing. Subsequent cooling and rewarming may cause irreversible damage to cell viability and structural integrity [7,8,9], thus allografts lose their toughness and elastic properties [10]. Up-to-date there are no guidelines that would describe ideal way of cryopreservation and subsequent thawing in order to obtain allografts of the highest possible quality and durability.

### Ethical statement

All the allografts were harvested in the operation theater in patients that were organ donors and were pronounced “clinically dead” with compliance to the Czech Republic’s transplants laws.

All 3 clinical departments (2^nd^ Department of Cardiovascular Surgery, General University Hospital, Prague, Czech Republic; Transplant Center & Department of Cardiac Surgery, University Hospital Motol, Prague, Czech Republic; Tissue Bank, Faculty Hospital Hradec Kralove, Charles University—Faculty of Medicine in Hradec Kralove, Hradec Kralove, Czech Republic) have approved regulations dealing with experimental work on cryopreserved human tissues. These regulations were approved by the particular Ethical Committee. Individual consents for the use of tissue are not available as the allografts are not stored under the name of the donor, the individual donor cannot be traced and the experiments were performed only on allografts that were removed from tissue bank as ‘‘unsuitable for patient transplant” (usually when their suitability for transplantation expired after the accepted time).

This study was reviewed and approved by the Ethical Committee of General University Hospital, Prague Czech Republic.

### Allografts harvest and characteristics

Basic allograft characteristics for Thawing Protocol 1 (thawing at a room temperature at 23°C) are summarized in Table 1. Basic allograft characteristics for thawing protocol 2 (thawing in a water bath at +37°C) are summarized in Table 2.

**Table 1:** Thawing protocol 1: Basic allografts characteristics.

Gender	Donor Age	Aorta diameter/mm	ABO, RH Compatibility
Female	55	21	A+
Femlae	41	21	A+
Male	55	25	AB+
Female	56	24	A+
Male	57	27	B+
Male	59	28	O-

**Table 2:** Thawing protocol 2: Allografts basic characteristics.

Gender	Donor Age	Aorta diameter/mm	ABO, RH Compatibility
Male	34	21	A-
Female	51	24	B+
Male	44	24	B+
Male	44	25	O-
Male	42	27	AB+
Female	37	27	A+

### Allograft processing cryopreservation protocol

All human ARA underwent an initial decontamination according to the standard protocol of the tissue bank. Afterwards, all allografts are stored in an antibiotic cocktail comprising of Cefuroxime 0.2 mg/ml + Piperacilline 0.2 mg/ml + Netilmicine 0.1 mg/ml + Fluconazole 0.1 mg/ml in tissue culture nutrient medium E 199 for 24 hours at + 37°C. Subsequently, all ARA were moved into the cryoprotectant solution in sterile laminar flow cabinet, and packed by double layer technique (sealed in Gambro Hemofreeze bags, NPBI BV, Gambro, The Netherlands). Cryprotectant used was 10% dimethylsulfidoxide in nutritional source for cell culture E 199. After the packaging was completed, all ARA were cooled at a controlled rate of -1°C/min from + 10°C to -60°C, then rapidly cooled and stored in a cryocontainers with liquid phase of liquid nitrogen at-196°C.

### Thawing protocols

Experimental work was based on investigating 12 cryopreserved CHARAs. CHARAs were randomly divided into two groups, each group consisting of six samples. All allografts were thawed in their original packaging (packed by double layer technique and immersed in 10% dimethylsulfidoxide). Two thawing protocols were tested:

- Protocol 1: six cryopreserved human ARAs thawed at a room temperature at 23°C. Thawing times were as follows: minimum 2hr 49 min, maximum 4hr 5 min, with median of 3hr 19 min
- Protocol 2: six cryopreserved human ARAs were placed directly into a water bath at +37°C. Thawing times were as follows: minimum 26 minutes, maximum 41minutes, with median of 32 min

The time variability in both thawing protocols was given by the different allografts sizes (Tables 1 and 2), as well as different amounts of cryoprotectant used for each allograft during the cryopreservation process. After all the samples were thawed, part of an aortic root arterial wall was dissected from each aortic root, fixated in a 4% formaldehyde solution and sent for electron microscope testing.

### Microscopic slide preparation

After the thawing protocols were completed, a sample of aortic root arterial wall was carefully dissected from each specimen (the arterial wall of non-coronary sinus was harvested from each allograft). After the samples were collected, they were fixed in Baker’s solution. Each sample was divided into 5–10 mm sub-samples. Samples were mounted on convex polystyrene casts with hedgehog like spines. All samples were washed in distilled water for 5 min, and dehydrated in a graded ethanol series (70, 85, 95, and 100%) for 5 min at each level. The tissue samples were then immersed in 100% hexamethyldisilazane (CAS No. 999-97-3; Fluka Chemie AG, Buchs, Switzerland) (HMDS) for 10 minutes and air dried in an exhaust hood at room temperature.

Processed samples were mounted on stainless steel stubs, coated with gold, and stored in a desiccator until they were studied and photographed by Electron Microscope on scanning mode operating at 25 kV—BS 301. A special scoring system (from 1 to 6) was introduced in order to analyse morphological changes of the arterial wall of aortic root under the electron microscope: 1. morphologically intact endothelium—putative physiological changes are not reflected in the superficial morphology of endothelial cells, 2. confluent endothelium with structural inhomogeneity—irregularities in the form of individual cells and changes of their membranes are detectable, 3. disruption of intercellular contacts—continuity of endothelial coverage is lost, endotheliocytes shrink while still adhering to basal membrane, 4. separation of endothelial cells—endotheliocytes separate from the basal lamina. Initially they protrude by their intercellular edges into the lumen, 5. complete loss of endothelium—denudation of the endothelial covering with the basal lamina exposed, 6. damage of subendothelial layers—the valvular surface is covered only by remnants of basal membrane, the fiber structure of the lamina fibrosa and the lamina ventricularis may be dissolved [11] (Table 3).

**Table 3:** Scoring system for morphological sample analysis.

Score	Morphology
1	morphologically intact endothelium—putative physiological changes are not reflected in the superficial morphology of endothelial cells
2	confluent endothelium with structural inhomogeneity—irregularities in the form of individual cells and changes of their membranes are detectable
3	disruption of intercellular contacts—continuity of endothelial coverage is lost, endotheliocytes shrink while still adhering to basal membrane
4	separation of endothelial cells—endotheliocytes separate from the basal lamina. Initially they protrude by their intercellular edges into the lumen
5	complete loss of endothelium—denudation of the endothelial covering with the basal lamina exposed
6	damage of subendothelial layers—the valvular surface is covered only by remnants of basal membrane, the fiber structure of the lamina fibrosa and the lamina ventricularis may be dissolved.

## Results

**Fig 1:**
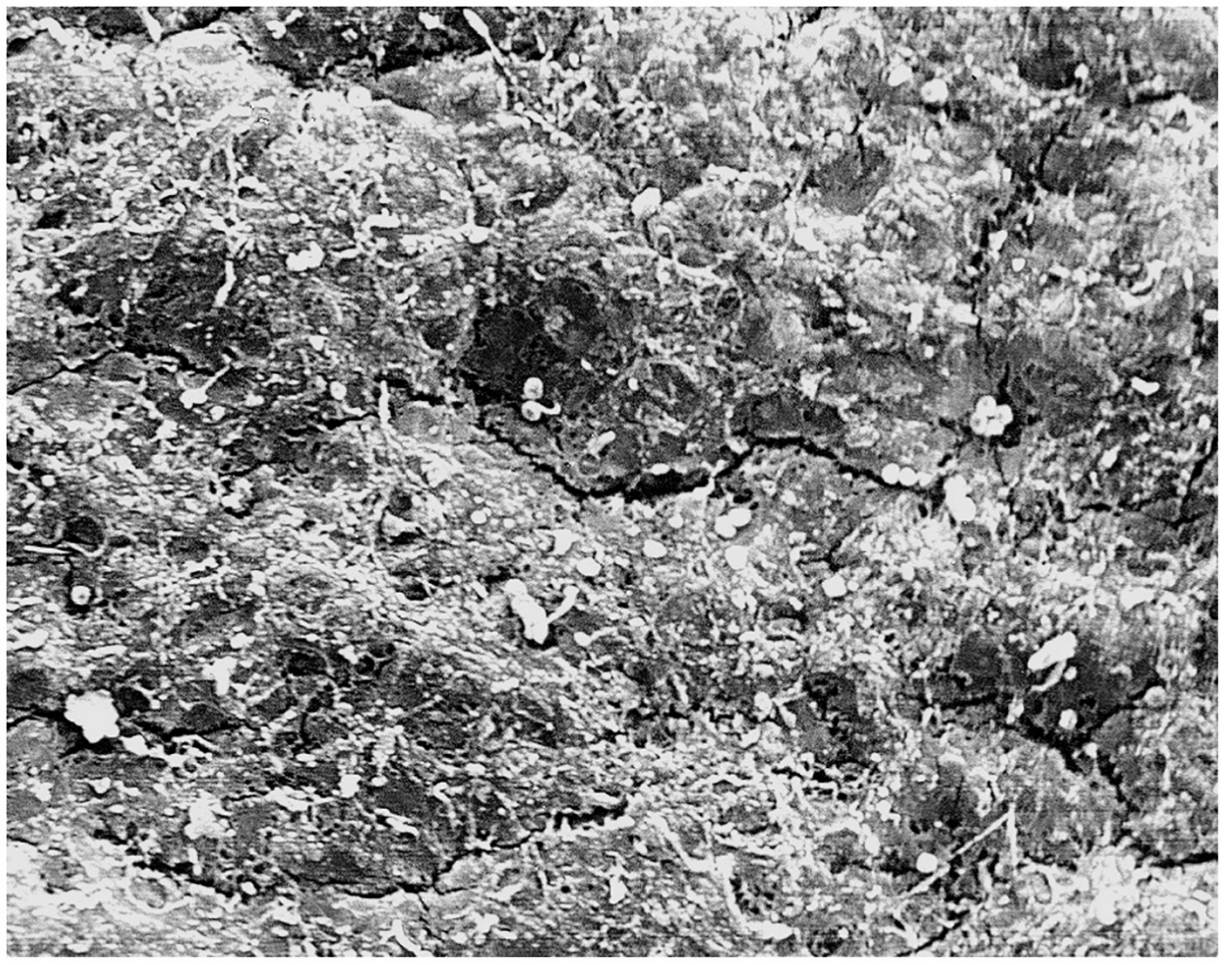
Aortic root arterial wall (Thawing protocol 1)—Loss of the endothelium exposing the basal lamina, damage to the subendothelial layers with randomly dispersed circular defects and micro-fractures. (Magnification- 560x).

**Fig 2:**
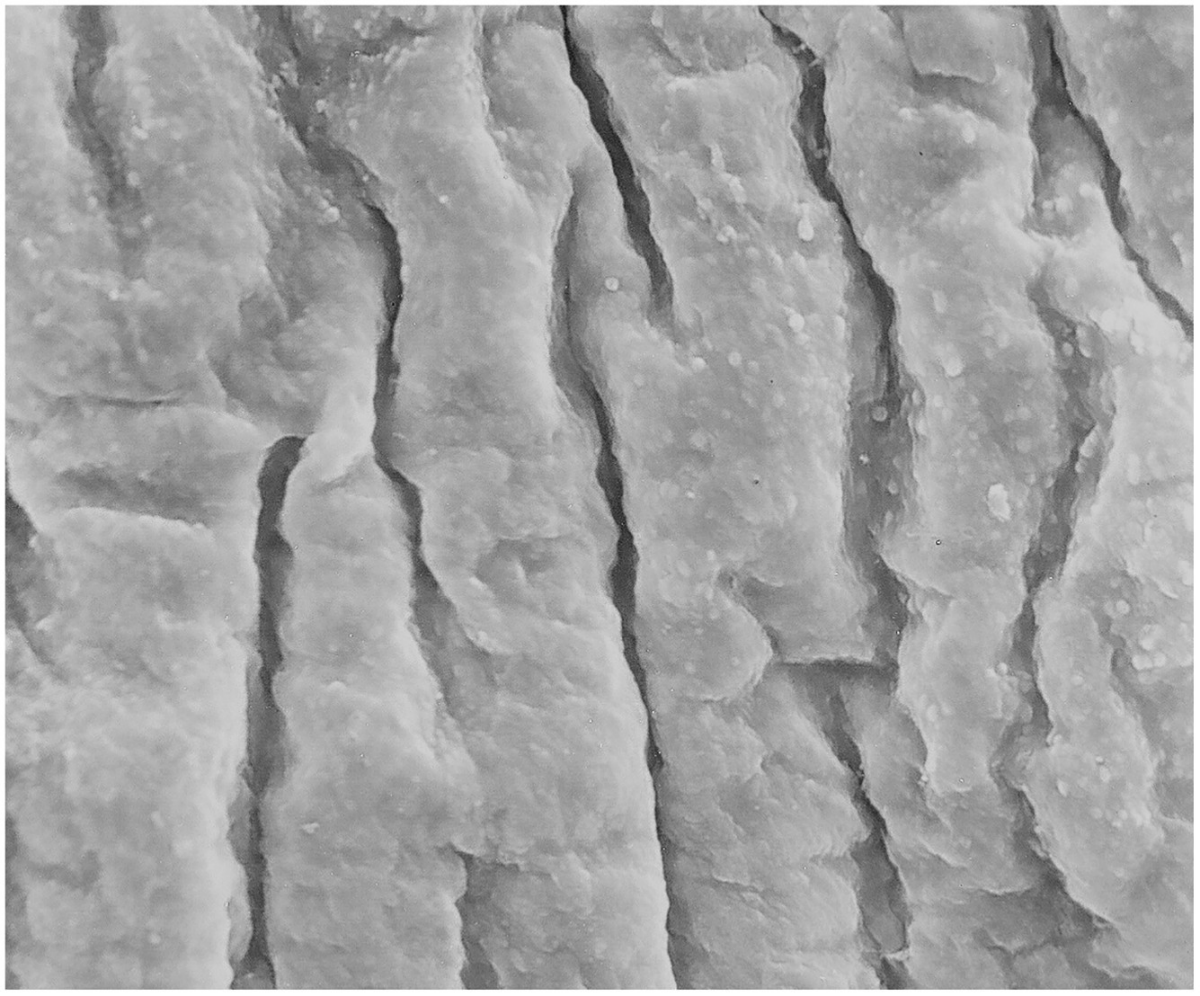
Loos of the endothelium from the luminal surface, longitudinal corrugations in the direction of blood flow caused by smooth muscle cells contractions in the tunica media. (Magnification- 520x).

**Fig 3:**
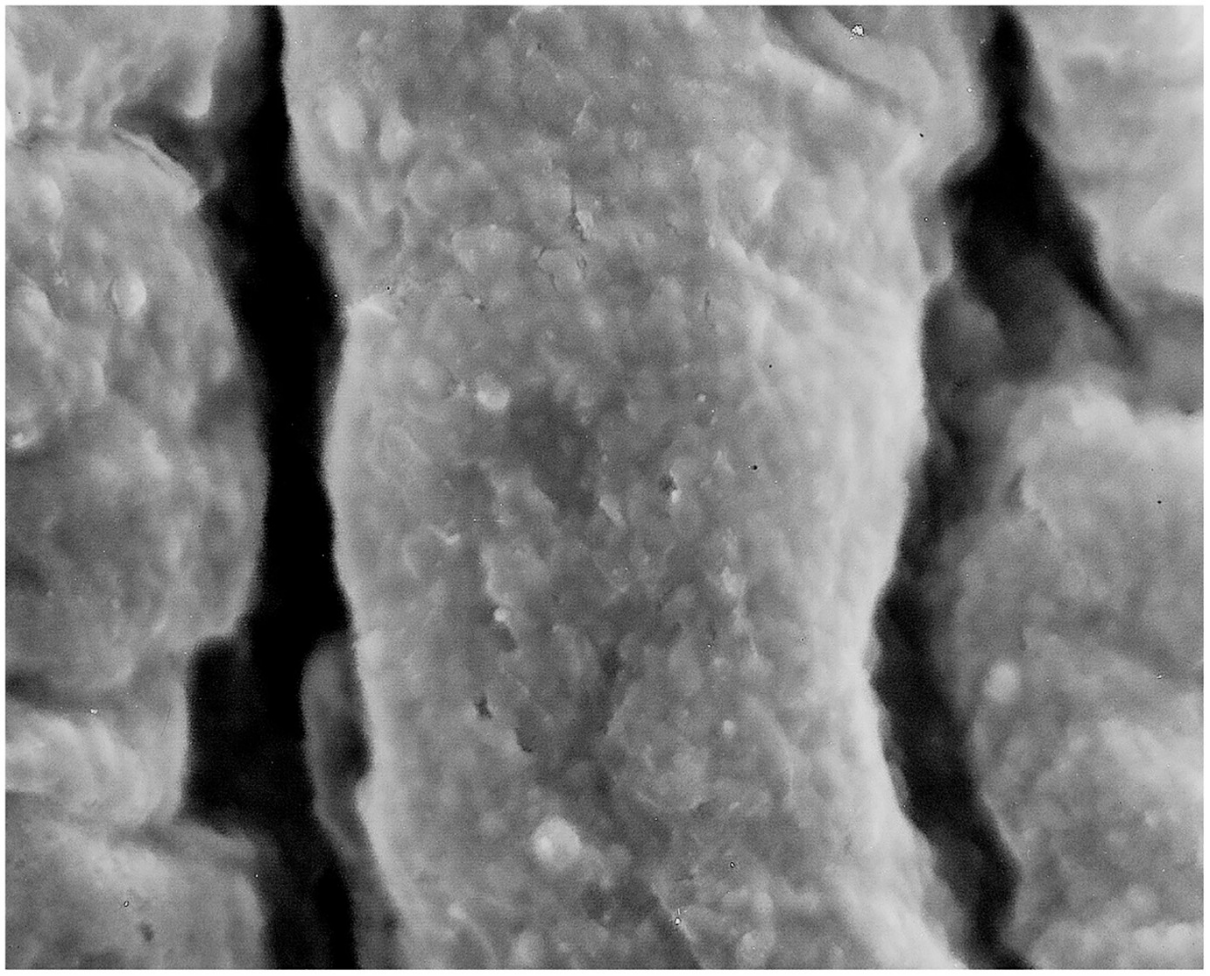
Loos of the endothelium from the luminal surface, longitudinal corrugations in the direction of blood flow caused by smooth muscle cells contractions in the tunica media with frequent fractures in the subendothelial layer. (Magnification 1700x).

Histological analysis of the aortic root arterial wall was as follows:

- Thawing protocol 1(thawing at a room temperature +23°C): All 6 (100%) samples showed loss of the endothelium exposing the basal lamina, damage to the subendothelial layers with randomly dispersed circular defects and micro-fractures. Arterial wall was not contracted (Fig 1). Furthermore, 4 (66%) samples showed no damage to the basal lamina (Score 5), 1(17%) sample showed minimal damage to the basal lamina (Score 5), and 1 (17%) sample showed severe damage to the basal lamina (Score 6).
- Thawing protocol 2 (water bath at +37°C): All 6 (100%) samples showed loss of endothelium from the luminal surface, longitudinal corrugations in the direction of blood flow caused by smooth muscle cells contractions in the tunica media with frequent fractures in the subendothelial layer (Figs 2 and 3). Furthermore, 5 (83%) samples showed severe basal lamina damage (Score 6), and 1(17%) sample showed no basal lamina with a severe damage to the internal elastic lamina (Score 6).

## Discussion

Over the past four decades, cryopreservation of arterial allografts had shown inconsistent results in long term graft durability. In order to understand the changes that occur during cryopreservation and thawing of allografts, microscopic and immuno-histological techniques are used in order to determine structural and functional changes. One of the most important experimental works performed by M' Bengue-Gaye A et al. on a rabbits carotid arteries clearly showed the effects of cryopreservation on allografts [12]. It had been clearly demonstrated that allograft processing including cryopreservation and thawing are crucial in determining cryopreserved “muscular arteries” allografts durability and clinical performance [13,14].

Immunological reactions caused by cryopreserved arterial allografts are very complex and not fully understood. Arterial allografts are immunogenic as they induce anti-HLA antibody response in the recipient, thus causing graft rejection. The contribution of anti- HLA class I antibody to the structural allografts degradation is not clear [15]. Cryopreserved aortic root allografts are immunogenic, HLA-ABC and HLA—DR antigen molecules can be identified on the aortic wall and aortic valve leaflet [16]. Rodriguez M et al. proved that slow thawing protocol causes the immune response to subside when compared to fresh arterial allografts [17].

Although cryopreserved aortic root allograft transplants are associated with outstanding hemodynamics, low thromboembolic events and low risk of endocarditis, the biggest concern is their long-term durability and subsequent risk of reoperation based on the allograft structural degradation related to the degradation of valvular leaflets, thus leading to aortic valve insufficiency [18,19,20]. Progressive allograft atherosclerosis caused by a chronic rejection is one of the major factors that leads to a short-term graft survival with characteristic diffuse intimal thickening, migration and proliferation of smooth muscle cells [21,22]. Allograft vasculopathy is caused by an injury inflicted as a response to the transplantation, causing endothelial dysfunction and intimal hyperplasia development, thus contributing to the development of allograft atherosclerosis. Experimental work by Xu Ziagiang et al. on rat model showed that atherosclerosis—multifactorial complex associated with immunologic and non- immunologic risk factors can be controlled into some extent [23]. Calcification of the aortic root arterial wall is another major preconditioning for aneurysm formation, and is the main cofactors in systemic calcific embolization [24]. Another key aspect is the change of toughness and biaxial tensile properties of cryopreserved arterial allografts that lead to early aneurysm formation and ruptures in high pressure arterial circulation [10].

Cryopreserved aortic root allografts are widely used in a cardiac surgery as a means of surgical treatment of a prosthesis infection. Based on short and mid-term results, cryopreserved aortic root allografts showed acceptable results with a low risk of reinfection. Patients that receive cryopreserved allografts require a long-term follow-up for both infection and implant durability [25,26]. Despite the advances in understanding morphological and functional changes that are caused by cryopreservation and subsequent thawing, there are still many immunological processes that are not fully understood and are in the need of further investigation [15,27, 28, 29].

### Conclusion

Based on our results, we have demonstrated that all the samples of CHARAs thawed at the room temperature showed smaller overall structural damage to the arterial and no smooth muscle cell contraction in tunica media when compared to the samples thawed in a water bath. Thawing at a room temperature seems to be gentler and does not lead to so severe damage of the CHARAs arterial wall. Consequently, based on our histological findings, we can conclude that the samples thawed at a room temperature should be in theory of higher quality compared to samples thaw at water bath, thus they should be more suitable for transplantation. Our finding shows the importance of technical aspects even for well-established surgical procedures.
